# Illicit substance use after release from prison among formerly incarcerated primary care patients: a cross-sectional study

**DOI:** 10.1186/s13722-019-0136-6

**Published:** 2019-02-19

**Authors:** Adam Chamberlain, Sylviah Nyamu, Jenerius Aminawung, Emily A. Wang, Shira Shavit, Aaron D. Fox

**Affiliations:** 1 Albert Einstein College of Medicine, Bronx, NY 10461 USA; 2 Mount Sinai St.Luke’s and Mount Sinai West Hospitals, New York, NY 10019 USA; 3 Yale University School of Medicine, New Haven, CT 06520 USA; 4 University of California-San Francisco, San Francisco, CA 94103 USA; 5 Montefiore Medical Center, 111 E. 210th Street, Bronx, NY 10467 USA

**Keywords:** Illicit substance use, Incarceration, Primary care, Transitions Clinics

## Abstract

**Background:**

More than 80% of people in jail or prison report having used illicit substances in their lifetimes. After release from incarceration, resumption of substance use carries risks, including parole revocation, exacerbation of mental health conditions, transmission of infectious diseases, and drug overdose.

**Methods:**

This cross-sectional study used baseline data from the Transitions Clinic Network (TCN, www.transitionsclinic.org), a multi-site prospective longitudinal cohort study of post-incarceration medical care. We investigated substance use among adults, with at least one chronic health condition or age ≥ 50 years, who had been recently released from incarceration and initiated care at a TCN site. Our primary outcome was any self-reported illicit substance use (heroin or other opioids, cocaine, cannabis, amphetamines, hallucinogens, MDMA, or illicit use of prescription medications) following release from incarceration. Alcohol use post-release was a secondary outcome. Using multivariable logistic regression, we also explored factors associated with illicit substance use.

**Results:**

Among 751 participants, median age was 47; participants were mostly male (85%), non-white (47% black, 30% Hispanic), and on parole (80%). The proportion of participants reporting any illicit substance use and any alcohol use soon after release from incarceration was 18% and 23%, respectively. In multivariable regression, variables significantly associated with post-release illicit substance use were male gender (aOR = 3.91, 95% CI: 1.73–8.81), housing with friends or family (aOR = 3.33, 95% CI: 1.20–9.28), years incarcerated during latest prison term (aOR = 0.93, 95% CI: 0.89–0.98), weeks elapsed before engagement with TCN (aOR = 1.07, 95% CI: 1.03–1.10), being on parole (aOR = 0.58, 95% CI: 0.34–0.99), and having a drug use disorder (aOR = 2.27, 95% CI: 1.40–3.68).

**Conclusions:**

Among individuals seeking medical care after release from incarceration, self-reported substance use was lower than previously reported estimates of post-incarceration substance use. Known risk factors, such as male gender and having a drug use disorder, were associated with illicit substance use, as were novel risk factors, such as less supervised housing. Though illicit substance use post-incarceration can carry severe consequences, treatment and surveillance interventions should be targeted toward individuals with greatest risk.

## Background

The criminal justice system has an exceptionally broad reach in the United States. At any one time, over 2 million people are incarcerated with 1.3 million in state prisons, 630,000 in local jails, nearly 200,000 in federal prison, and 40,000 in immigration detention centers [1]. Problematic substance use is common among this population with more than 80% of people in jail or prison reporting having used illicit substances in their lifetimes [1, 2]. The majority of people in jails (53%), state prisons (56%), or federal prisons (50%) met DSM-IV criteria for a substance use disorder (SUD) at the most recent national surveys [3–5]. Additionally, according to a report by the Substance Abuse and Mental Health Services Administration (SAMHSA), 19% of males on probation (i.e. sentenced and serving time in the community) aged 18–49 had a drug use disorder (DUD) and over a quarter had an alcohol use disorder (AUD) in 2012 [6]. However, few incarcerated individuals receive evidence-based SUD treatment, and substance use often continues during and after incarceration [7, 8].

During incarceration, less than 20% of individuals with SUDs receive formal treatment. Pharmacotherapies, such as methadone maintenance treatment for opioid use disorder, are rarely offered in correctional settings; even when available, only a small fraction of eligible individuals access treatment [9]. Addressing substance use and SUDs in the criminal justice population will require additional attention and new approaches.

Substance use disorders are chronic relapsing conditions. Even those who stop substance use during incarceration may resume use post-release, which introduces several risks [9]. Numerous studies have documented greatly elevated risk of death when people are released from jail or prison with the leading cause of death being drug overdose [10, 11]. People in jail or prison who resume injecting drugs post-release are also at risk for transmitting viral infections, such as HIV or Hepatitis C Virus [12]. Because of the co-occurrence of SUDs and mental health conditions, post-release substance use may also worsen mental health status and prevent engagement in needed medical care [13]. Additionally, post-release substance use can lead to re-incarceration. Formerly incarcerated individuals with SUDs or substance-related criminal charges are more likely to be re-incarcerated than those without substance involvement [14, 15]. Qualitative research suggests that substance use post-release may be due to poor mental health, environmental exposures (e.g., substance-using peer groups), or life stressors related to community re-entry, such as challenges finding work and stable housing [16–18]. Additional research is needed to better understand substance use among people who have been released from jail or prison.

Post-release substance use can carry risk even when individuals do not have SUDs. General conditions of parole prohibit the use or possession of a controlled substance [19]. Drug testing procedures for individuals on parole vary depending on state regulations, but post-release substance use could lead to parole violations [20]. In New York State, for instance, any possession of drug paraphernalia or use or possession of a controlled substance without medical authorization may result in parole revocation [21]. Therefore, research on substance use post-release should include individuals both with and without SUD diagnoses.

While substance use and SUDs are common prior to incarceration, there is a dearth of data on the rate of substance use post-release. One systematic review highlighted that most studies of SUD care post-incarceration have reported criminal justice outcomes (e.g., re-incarceration) instead of substance use outcomes. [22] Additionally, existing research has focused on cohorts of individuals in SUD treatment who likely carry the greatest risk of substance use resumption [23–26]. For example, a study assessing the efficacy of a therapeutic community treatment program for formerly incarcerated individuals with SUDs found that 79% of participants in the treatment group resumed illicit drug use within 5 years post-release [24]. A clinical trial assessing opioid use disorder treatments at prison release found that more than three-quarters of the study arm that received pre-release counseling resumed heroin use at 3 months post-release [25]. A cohort study tracking individuals released from jail with varying levels of substance use found that 43% had at least one substance dependence symptom 1 year post-release; over a quarter (28%) reported cocaine use and a third (33%) reported cannabis use [26]. These data highlight the chronicity of severe SUDs; however, it also possible that individuals with less severe substance use do cut down or stop substance use post-release, which has implications for monitoring and service provision.

In this study, we investigated substance use in a diverse group of individuals who were recently released from incarceration. These data could improve generalizability of knowledge regarding substance use resumption post-incarceration, which to date has been mostly derived from individuals receiving SUD treatment. We also explored factors associated with substance use following release from incarceration. Understanding the trajectory of substance use following release from incarceration for people with and without SUDs can guide the development of targeted interventions for those at greatest risk of poor substance use-related outcomes.

## Methods

This cross-sectional study utilized baseline data from the Transitions Clinic Network (TCN, www.transitionsclinic.org), a multi-site prospective longitudinal cohort study of post-incarceration medical care.

### Setting

The TCN is a national consortium of 24 primary care centers that serve the health needs of individuals returning from incarceration. The current study derives from 13 sites that participated in the longitudinal cohort study. Multi-disciplinary health care teams at each site include community health workers (CHWs) who have a history of incarceration and have been trained in health education, health system navigation, and motivational enhancement. CHWs link individuals released from prison or jail to primary care at TCN sites. Other features of TCN sites include: providers who have received training in best practices in caring for individuals with criminal justice involvement; ability to provide or refer for mental health and SUD services; and collaboration with social service providers, including housing, employment, and legal aid agencies. Individual TCN sites have been described in more detail [27, 28]. Many sites are part of integrated health systems with specialty SUD services, but most patients were referred to TCN sites to initiate primary care.

### Participants

All new patients at TCN sites seen between May 2013 and February 2015 were screened for inclusion in the cohort study. Referrals of recently released individuals with chronic conditions came from three main sources: correctional agencies—specifically, prisons and parole and probation offices; community agencies, such as social service agencies and community-based organizations; and traditional sources such as other clinicians or self-referral from patients [29]. Inclusion criteria were: (1) recent release from prison (within 6 months); (2) presence of at least one chronic health condition warranting primary medical care, including SUD as a chronic health condition, or age equal to or greater than 50 years old; (3) ability to provide informed consent in English or Spanish; and (4) a plan to live in the area near the TCN program site for the duration of the study. Patients who planned to return to a previous primary care provider were excluded. All participants provided written informed consent, and data was protected by a certificate of confidentiality from the National Institutes of Health.

### Data collection

We used data from baseline surveys for all participants of the TCN cohort study. Surveys were administered by trained research staff in person or via telephone. Data were stored in an online HIPAA-compliant portal and relevant clinical information was provided to primary care providers to facilitate medical care. Data included sociodemographic factors, self-reported incarceration history, past medical, mental health, and substance use history and treatment.

### Measures

#### Substance use

Our primary outcome variable was any self-reported illicit substance use following release from incarceration. Use of each of the following substances post-release was assessed: heroin or other opioids, cocaine, cannabis, amphetamines, hallucinogens, MDMA, or illicit use of prescription medications. We assessed lifetime use, use since release, and frequency of use, but for this analysis, any use of any of these substances post-release was considered illicit substance use. A secondary outcome was any self-reported alcohol use assessed based on frequency and quantity of use post-release (i.e., the number of days per week and standard drinks per day when alcohol was consumed). Participants also self-reported whether they had ever been diagnosed with a SUD. For this analysis, we differentiated between presence of a drug use disorder (DUD) and alcohol use disorder (AUD).

#### Substance use disorder treatment

Participants who self-reported a DUD or AUD were also assessed for receipt of DUD and AUD treatment, respectively. Participants self-reported whether they received treatment while they were incarcerated and at the time of the survey. Participants indicated the type(s) of treatment they received by choosing from a list with the following options: Alcoholics Anonymous/Narcotics Anonymous (AA/NA) or self-help groups; pharmacotherapy; one-on-one counseling; or other, where participants could give free text responses. Participants were able to choose more than one type of treatment.

#### Psychiatric diagnoses

Participants were asked about prior psychiatric diagnoses, including SUDs. They also self-reported diagnoses of depression, bipolar disorder, post-traumatic stress disorder (PTSD) and schizophrenia. In addition to self-report, surveys included validated screening instruments for PTSD and Depression (Primary Care PTSD screen and the Patient Health Questionnaire) [30, 31].

#### Criminal justice history

Participants self-reported criminal justice involvement in several ways: time spent incarcerated during their most recent prison term, lifetime arrest and conviction counts, current parole/probation status, restricted incarceration status and the amount of time that had passed since release from incarceration.

#### Covariates

Other data collected included sociodemographic factors (age, binary gender, race/ethnicity, education, marital status), employment status and history (including employment, access to cash, benefits and other income sources), food security, housing security (concern for becoming homeless within 4 weeks), and housing type. The survey prompted participants to choose between eight different housing types, which we used to create four categories: unstable (street homeless; living in a shelter or single room occupancy hotel), institutional (drug treatment facility or other type of residential facility), “doubling-up” (staying with friends or family), and rent/own (renting or owning one’s own apartment or house).

### Data analysis

First, we conducted descriptive statistics to assess the characteristics of the cohort. Next, we determined the proportion of participants reporting post-incarceration illicit substance use. Frequencies and proportions were assessed separately for each substance, and for the composite measure of any illicit substance use, which did not include alcohol use. Next, we built a multivariable logistic regression model with any illicit substance use as the outcome measure (dichotomous, yes/no). The entire sample (i.e., individuals with and without prior DUD or AUD) was included in the regression model. For model building, we explored factors associated in bivariate testing with post-release illicit substance use by using Chi square, student’s *T* test or Mann–Whitney tests. Covariates that were associated with post-release substance use (p < 0.10) were then included in the multivariable logistic regression model. After bivariate testing, the covariates that were included in the final regression model were: age, gender, race/ethnicity, housing type, time incarcerated at latest prison term, time to engagement with TCN site, parole status, depression, bipolar disorder, and DUD diagnosis. Finally, we performed sensitivity analyses to test the robustness of our multivariable regression model. In the first, we restricted the sample to only participants with a DUD diagnosis and repeated the modeling approach. Our goal was to determine whether factors associated with post-release illicit substance use differed between participants with and without a prior DUD diagnosis. In the second, we restricted the sample to only participants on parole and again repeated the modeling approach without parole status as an independent variable. Our goal was to determine whether overall substance use and associated covariates changed when excluding participants who were not monitored by parole.

## Results

Of the 751 participants who completed the TCN baseline survey, the median age was 47, participants were mostly male (85%), non-white (47% black, 30% Hispanic), and had not graduated from high school (59%). Participants were most commonly on parole (80%), lived in institutional housing (39%), and unemployed (92%). The median time incarcerated during participants’ most recent prison term was 4 years (*Interquartile range*: 2–8 years). The median time from prison release to engagement at a TCN site was 5 weeks (IQR: 2–9 weeks). Among clinical factors, slightly less than half of all participants reported a prior diagnosis with depression (46%) or a drug use disorder (45%) (see Table 1).

**Table 1:** Demographic and clinical characteristics of 751 participants who received medical care following release from prison

Demographic characteristic	Reported any substance use since release (%) (n = 134)	Did not report any substance use since release (%) (n = 617)	Total n (%) (n = 751)	p-value
Age, median (IQ range)	45 (35–51)	48 (39–54)	47 (38–53)	*< 0.01*
Male	123 (92)	517 (84)	640 (85)	*0.02*
Race/ethnicity				
Hispanic	36 (27)	191 (31)	227 (30)	NS
Non-Hispanic black	72 (54)	280 (45)	352 (47)	0.08
Non-Hispanic white	19 (14)	115 (19)	134 (18)	NS
Other	7 (5)	31 (5)	38 (5)	NS
Graduated high school	50 (38)	255 (42)	305 (41)	NS
Receive employment earnings	7 (5)	54 (9)	61 (8)	NS
Receive any income	61 (46)	316 (51)	377 (50)	NS
Housing				
Unstable	44 (33)	148 (24)	192 (26)	*0.04*
Institutional	25 (19)	266 (42)	291 (39)	*< 0.01*
“Doubling up”	58 (43)	152 (25)	210 (28)	*< 0.01*
Rent/own	7 (5)	50 (8)	57 (7)	NS
Years incarcerated during latest prison term, median (IQ)	2 (1–4)	4 (2–9)	4 (2–8)	*< 0.01*
Weeks to TCN engagement, median (IQ)	7 (3–14)	4 (2–9)	5 (2–9)	*< 0.01*
Current parole	95 (72)	501 (82)	596 (80)	*< 0.01*
Reported diagnoses				
Depression (N = 683)	71 (59)	240 (43)	311 (46)	*< 0.01*
Bipolar (N = 677)	42 (36)	141 (25)	183 (27)	*0.02*
PTSD (N = 680)	23 (19)	100 (18)	123 (18)	NS
Schizophrenia (N = 684)	25 (20)	84 (15)	109 (16)	NS
Drug use disorder (N = 689)	74 (60)	236 (42)	310 (45)	*< 0.01*
Alcohol use disorder (N = 695)	34 (27)	171 (30)	205 (30)	NS
Received SUD treatment in prison (N = 362)	53 (65)	201 (72)	254 (70)	NS

Italic = statistically significant

The proportion of participants reporting any illicit substance use and any alcohol use soon after release from incarceration was 18% and 23%, respectively. The 134 participants who reported any illicit substance use post-release differed in demographic, social and clinical characteristics from those without illicit substance use. In bivariate analysis there were significant associations between post-release illicit substance use and younger age, male gender, not being on parole, housing status, psychiatric diagnoses, incarceration history and time to engagement at a TCN site. Illicit substance use was positively associated with unstable housing and doubling up and negatively associated with institutional housing. Spending fewer years incarcerated during the most recent prison term was associated with post-release illicit substance use. Reporting a prior diagnosis of depression, bipolar disorder or DUD was also associated with post-incarceration illicit substance use (Table 1).

In regards to post-release illicit substance use, cannabis use was most common with 12% of participants reporting post-release cannabis use. Fewer participants reported post-release cocaine or opioids use: 4% for each substance (see Table 2). Of participants with a DUD diagnosis, 67% reported receiving treatment during incarceration. The most common form of treatment reported was narcotics anonymous (61% of those who received treatment). Formal programs (20%), one-on-one counseling (20%), and pharmacotherapy (4%) were less commonly reported. One participant reported receiving art therapy.

**Table 2:** Post-release illicit substance use for 751 participants who received medical care following release from prison

Illicit substance use	Never* N (%)	No use since release* N (%)	Use post-release* N (%)
Cannabis (N = 744)	155 (21)	503 (68)	86 (12)
Cocaine (N = 746)	325 (44)	391 (52)	30 (4)
Heroin/Opioids (N = 740)	445 (60)	263 (36)	32 (4)
Amphetamine (N = 740)	547 (74)	184 (25)	9 (1)
Hallucinogens (N = 742)	581 (78)	157 (21)	4 (1)
MDMA (N = 625)	546 (87)	78 (12)	1
Illicit prescriptions (N = 715)	538 (75)	154 (22)	23 (3)
Any illicit drug Use (N = 751)	91 (12)	526 (70)	134 (18)

*Percentages are of participants who reported any substance use status for each separate substance

In the multivariable regression model several variables remained significantly associated with post-release illicit substance use, including male gender (aOR = 3.91, 95% CI: 1.73–8.81), housing with friends or family (aOR = 3.33, 95% CI: 1.20–9.28), time incarcerated during most recent prison term (aOR = 0.93, 95% CI: 0.89–0.98), weeks elapsed before engagement with TCN (aOR = 1.07, 95% CI: 1.03–1.10), being on parole (aOR = 0.58, 95% CI: 0.34–0.99), and having an DUD diagnosis (aOR = 2.27, 95% CI: 1.40–3.67) (see Table 3). In the first sensitivity analysis among those only with DUD, housing with friends or family was no longer significantly associated with illicit substance use, but the point estimate of the odds ratio remained similar to that of the full sample (aOR = 2.74, 95% CI: 0.65–11.56). Other variables maintained statistical significance. In the second sensitivity analysis, restriction of the sample to only those on parole was not found to affect which covariates in the multivariable model maintained significance.

**Table 3:** Factors associated with post-release illicit substance use

Independent variable*	Odds ratio	95% confidence interval
Age	0.98	0.96–1.00
Male	3.91	1.73–8.81
Black	1.31	0.82–2.09
Unstable housing	1.68	0.58–4.82
Institutional housing	0.81	0.28–2.35
Doubling up	3.33	1.20–9.28
Length of most recent incarceration (years)	0.93	0.89–0.98
Time to TCN engagement (weeks)	1.07	1.03–1.10
Parole	0.58	0.34–0.99
Depression	1.32	0.82–2.13
Bipolar disorder	1.62	0.97–2.71
Drug use disorder	2.27	1.40–3.68

*662 participants with complete data included in regression

## Discussion

In our cohort of individuals recently released from prison who initiated medical care at a transitions clinic, 18% reported illicit substance use between their prison release and first primary care appointment. In multivariable analysis, we found that post-release substance use was associated with expected risk factors such as drug use disorders, male gender, parole status, and time elapsed between release and the first medical encounter. Interestingly, housing status—specifically, living “doubled up” with friends or family members—had among the strongest association with post-release substance use, and this did not change when we excluded participants who were not monitored by parole (data not shown). Also, greater amount of time incarcerated at the latest prison term (in years) was associated with lower odds of post-release illicit substance use. These findings suggest areas, such as post-incarceration aftercare for drug use disorders or structured housing environments, where interventions could reduce the risk of post-release substance use and perhaps consequences of substance use.

Our findings add to the literature on substance use after prison release by focusing on a general population instead of only participants enrolled in SUD treatment. Our post-release illicit substance use incidence was at the low end of the range of previously published studies (18% vs. 22–88% at 3–6 months [22, 25, 32] and 70–95% at 1–3 years post-release [33–35]. Our study enrolled primary care patients who were released from prison, while prior studies mostly enrolled individuals enrolled in SUD treatment who are likely at highest risk of relapse. In our study, a history of drug use disorders was common (45%) and associated with post-release substance use, but even among those with a drug use disorder history, only 24% reported illicit substance use after the time of release. One study that is commonly cited in the scientific literature (338 times as per Google Scholar, searched on August 5, 2018) and policy reports, estimates that 95% of substance-involved people in prison will relapse to substance use post-release; however, the results should be interpreted with acknowledgement of the sampling frame, which selected for individuals with severe substance use disorders [33]. Our study also has limitations regarding generalizability (see below), but there is likely high variability in risk of substance use after release from incarceration.

Important factors that likely influenced our lower incidence of substance use were that we engaged participants soon after their release, most were monitored by parole, and our sampling strategy selected for a cohort that was older than prior studies. The median time elapsed between release and the first medical encounter among the TCN group was only 5 weeks. Many prior studies reported substance use over longer periods of time post-release. This is important for two reasons. First, it is plausible that substance use incidence is low in the first months after release, but then increases proportionally with time. This is consistent with our data, which shows that each additional week between release and the first medical encounter was associated with a 7% increased odds of substance use. This is also consistent with a 2004 prospective study following a general cohort of formerly incarcerated individuals that found illicit substance use rates of 22% at 4–6 months post-release [32]. Qualitative data highlight how recently incarcerated people with SUDs may express confidence and motivation to avoid substance use soon after release, but challenges during community reentry and accompanying emotional distress may lead to substance use [36]. Second, the majority of our participants were monitored by parole, which may have affected decisions around substance use. If participants’ parole monitoring included urine drug testing, this may have effectively discouraged illicit substance use. Data are conflicting about the types of monitoring practices that are most effective, but close supervision of substance use and certain and immediate consequences are considered best practices. [37] Third, the median age within our cohort was 47 years, and most national surveys in the United States suggest that incidence and prevalence of alcohol and drug use disorders decrease with age [38]. The prior studies cited above that reported post-release substance use had enrolled participants that were approximately 7–17 years younger than our cohort. Older age was not significantly associated with illicit substance use in our cohort, but selection of an older sample may have affected our low reported estimates of substance use. Engaging formerly incarcerated individuals in the early post-release period and capitalizing on parole’s influence on substance use could support abstinence from substance use.

Another interesting finding from our study is that housing status may also be associated with post-release substance use. Individuals who were “doubled up,” meaning living with friends or family members, were at higher risk of post-release substance use than those housed in other settings. Studies of housing in the post-release period generally measure the effects of institutional or supportive housing on substance use outcomes, which has demonstrated significant reduction in substance use associated with residence in supportive housing for sufficient time in the post-release period. [39–41] Individuals who are “doubled-up” with friends or family members may be at particularly high risk for illicit substance use due to lack of institutional support or exposure to acquaintances also using substances. Another important consideration is that participants living outside of institutional settings may have been under less drug testing surveillance, both from the program, but also from the state.

There were several limitations to our study. Our data comes from a cross sectional survey so we cannot make any statements about causality. Refusal to participate in the study was not systematically collected, which could affect generalizability of substance use estimates. The substance use outcomes are based on self-report, and a summary of data was shared with clinicians, so participants may have under-reported substance use. However, some studies with this population have demonstrated higher rates of substance use upon self-report in comparison to urine drug testing. [25] This study’s substance use outcomes also include cannabis, which may not be appropriate in states where it is currently legal, but is still important nationally as positive drug tests are a common reason for re-incarceration [42]. This was a secondary analysis, and our multivariable regression model was exploratory, so associations should be confirmed in studies specifically designed to test these hypotheses. Finally, the participants were older than most cohorts of formerly incarcerated individuals. Also, we only enrolled participants who engaged in primary care. Therefore, younger individuals and those who do not engage in medical care may have higher rates of substance use.

Due to the high volume of prison releases annually, high prevalence of SUD diagnoses in this population, and high recidivism rate, more studies are needed to understand substance use following release from incarceration. Our data suggests that overall substance use may be lower than expected post-release, but highlights some areas—such as less supervised housing—where substance use may be more common. Preventing negative consequences of substance use post-release should be a high priority for clinicians and policy-makers. Substance use education and treatment services should be available post-release and targeted to those with greatest treatment needs.

## Abbreviations

AUD: alcohol use disorder
CHW: community health worker
DUD: drug use disorder
PTSD: post-traumatic stress disorder
SAMHSA: Substance Abuse and Mental Health Services Administration
SUD: substance use disorder (includes both drug use disorder and alcohol use disorder)
TCN: Transitions Clinic Network
